# *PIK3CA* mutations are associated with increased tumor aggressiveness and Akt activation in gastric cancer

**DOI:** 10.18632/oncotarget.18770

**Published:** 2017-06-28

**Authors:** Ji-Won Kim, Hye Seung Lee, Kyung Han Nam, Soyeon Ahn, Jin Won Kim, Sang-Hoon Ahn, Do Joong Park, Hyung-Ho Kim, Keun-Wook Lee

**Affiliations:** ^1^ Department of Internal Medicine, Seoul National University Bundang Hospital, Seoul National University College of Medicine, Seongnam 13620, Korea; ^2^ Department of Pathology, Seoul National University Bundang Hospital, Seoul National University College of Medicine, Seongnam 13620, Korea; ^3^ Department of Pathology, Haeundae Paik Hospital, Inje University College of Medicine, Busan 48108, Korea; ^4^ Medical Research Collaborating Center, Seoul National University Bundang Hospital, Seoul National University College of Medicine, Seongnam 13620, Korea; ^5^ Department of Surgery, Seoul National University Bundang Hospital, Seoul National University College of Medicine, Seongnam 13620, Korea

**Keywords:** PIK3CA, mutation, AKT, expression, gastric cancer

## Abstract

*PIK3CA* mutations are frequent in gastric cancer. However, their pathological and clinical implications are still unclear. We analyzed the clinicopathological characteristics according to the *PIK3CA* mutation status of patients with stage IB–IV disease who underwent gastrectomy between May 2003 and Dec. 2005 (cohort 1; *n* = 302) and of those with stage IV disease who received gastrectomy between Jul. 2006 and Dec. 2012 (cohort 2; *n* = 120). *PIK3CA* mutations were detected in 40 patients (13.2%) in cohort 1. In these patients, *PIK3CA*-mutant tumors were more frequently located in the upper third of the stomach (*p* = 0.021) and significantly showed poorly differentiated histology (*p* = 0.018) and increased lymphatic (*p* = 0.015), vascular (*p* = 0.005), and perineural invasion (*p* = 0.026). In addition, these tumors showed significantly increased lymphocyte and neutrophil infiltration in cancer stroma (*p* < 0.001), Epstein–Barr virus positivity (*p* < 0.001), and microsatellite instability (*p* = 0.015). Cytoplasmic Akt expression was significantly increased in these tumors (*p* = 0.001). In cohort 2, *PIK3CA* mutations were identified in 15 patients (12.5%). *PIK3CA*-mutant tumors showed significantly increased vascular invasion (*p* = 0.019) and microsatellite instability (*p* = 0.041). In addition, cytoplasmic Akt expression was also significantly increased (*p* = 0.018). However, in both cohorts, *PIK3CA* mutations were not associated with the prognosis of patients. In conclusion, *PIK3CA* mutations were associated with increased tumor aggressiveness, especially in locoregional disease, and Akt activation in gastric cancer. Our data suggest that *PIK3CA*-mutated gastric cancer is a distinct disease entity, which might need a different therapeutic approach.

## INTRODUCTION

Gastric cancer (GC) is the fifth most common cancer and the third leading cause of death worldwide [1]. *PIK3CA* is the third most frequently mutated gene in GC [2, 3]. *PIK3CA* mutations are present in approximately 9%–12% patients with non-hypermutated tumors and 32% patients with hypermutated tumors.

Phosphatidylinositol 3-kinase (PI3K)/Akt signaling pathway is important in cancer cell proliferation and survival [4]. PI3K contains regulatory p85 and catalytic p110 subunits. *PIK3CA* encodes p110α, an isoform of the PI3K catalytic subunit. *PIK3CA* somatic mutations are frequently present in various human cancers such as liver, breast, colon, ovary, and GC [5, 6]. Mutant *PIK3CA* promotes the proliferation and invasion of human cancer cells [7] and confers resistance against HER2-targeted therapy for breast cancer [8, 9], thus warranting further investigation as a potential therapeutic target. However, only limited data on GC are available at present. Available evidence suggests that *PIK3CA* mutations in GC are more frequent in Epstein–Barr virus (EBV)-positive and MSI (microsatellite instability) subtypes than in the other subtypes [2, 3, 10, 11]. The most frequently detected *PIK3CA* mutation is H1047R in exon 20 especially in MSI subtype of GC [2, 12]. However, pathological or clinical implications of *PIK3CA* mutations are still unclear [13]. These unclear data might be attributed to the small sample size and the heterogeneous patient populations in previous studies. Moreover, as the detection of genetic alterations using next generation sequencing is more commonly conducted, targeted agents focusing on *PIK3CA* mutations are expected to be tested with high priority in metastatic GC. However, there has been no study focusing on clinicopathological characteristics of *PIK3CA* mutations in patients with stage IV GC. In addition, we still do not know whether *PIK3CA* mutations have the same clinicopathological implications on patients with locoregional disease and those with distant metastasis.

In this study, we aimed to elucidate the implications of *PIK3CA* mutations on detailed pathological characteristics and clinical outcomes in patients with GC using two independent cohorts consisting of patients with resected stage IB–IV GC and those with stage IV disease who underwent palliative resection.

## RESULTS

### Cohort 1

In all, 302 patients were consecutively enrolled in cohort 1. The male to female ratio was 202:100, and the median age was 61 years (range, 29–89 years). Of these, 273 patients (90.4%) had stage IB-III GC and 29 patients (9.6%) had stage IV GC. All the patients underwent curative or palliative gastrectomy.

**Figure 1:**
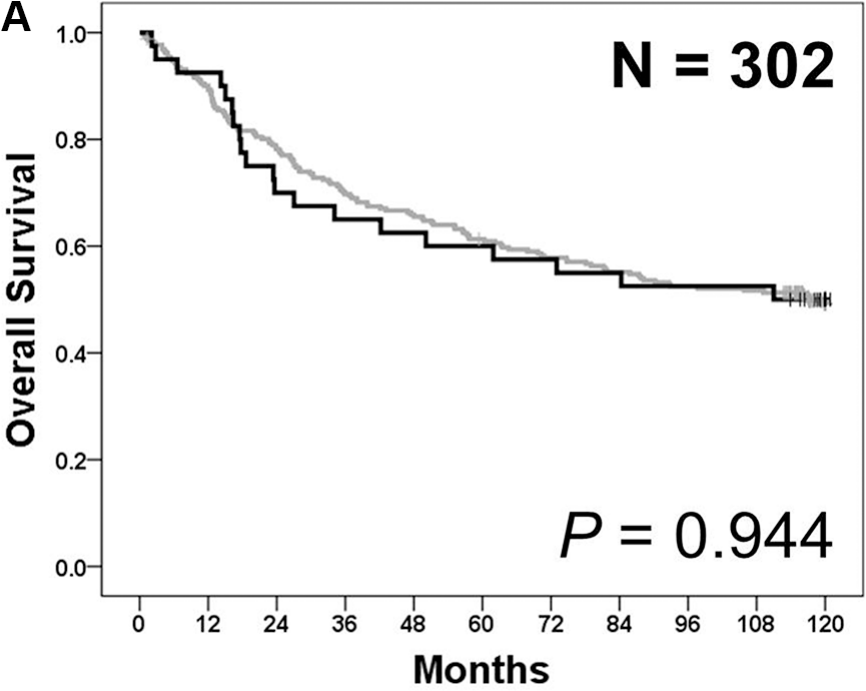

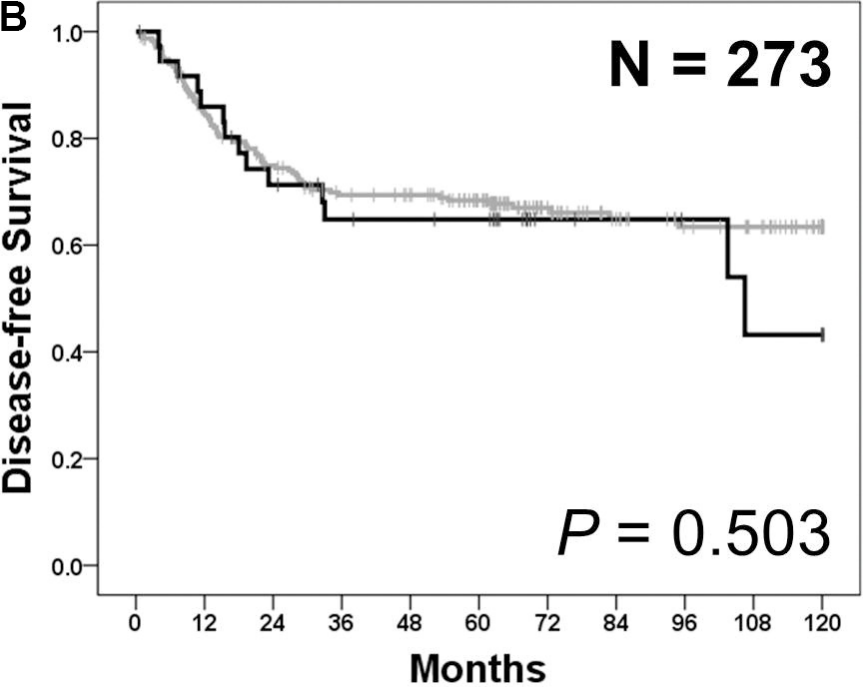
Survival analyses according to *PIK3CA* mutation status in cohort 1 (**A**) Overall survival was similar between patients with *PIK3CA*-mutant tumors (black) and those with *PIK3CA* wild-type tumors (gray) (*p* = 0.944). (**B**) In patients with stage I–III gastric cancer (*n* = 273), disease-free survival was similar between patients with *PIK3CA*-mutant tumors (black) and those with *PIK3CA* wild-type tumors (gray) (*p* = 0.503).

In all, 45 *PIK3CA* mutations were detected in 40 patients (13.2%), with E545X in 22 patients (7.3%), E542K in 10 patients (3.3%), H1047X in 7 patients (2.3%), Q546X in 2 patients (0.7%), N345K in 2 patients (0.7%), R88Q in 1 patient (0.3%), and C420R in 1 patient (0.3%) (Supplementary Table 1). The clinical and pathological characteristics of cohort 1 according to their *PIK3CA* mutation status are compared in Table 1. *PIK3CA*-mutant tumors were more frequently located in the upper third of the stomach (36.8% *vs.* 17.7%; *p* = 0.021) and showed advanced T stage (pT4: 52.5% *vs.* 32.8%; *p* = 0.018). Thus, total gastrectomy was more frequently conducted in patients with *PIK3CA*-mutant tumors than in those with *PIK3CA* wild-type tumors. *PIK3CA*-mutant tumors significantly showed poorly differentiated histology (72.5% *vs.* 46.2%; *p* = 0.018). Further, use of Lauren classification system showed that most *PIK3CA*-mutant tumors in cohort 1 were not intestinal-type tumors (15.0% *vs.* 35.5%; *p* < 0.001). *PIK3CA*-mutant tumors also showed increased lymphatic (92.5% *vs.* 75.2%; *p* = 0.015), vascular (35.0% *vs.* 16.4%; *p* = 0.005), and perineural invasion (72.5% *vs.* 53.8%; *p* = 0.026). Moreover, these tumors showed increased lymphocytic or neutrophilic infiltration in cancer stroma (55.0% *vs.* 18.3%; *p* < 0.001), yielded significant positive results for EBV-encoded small RNA *in situ* hybridization (EBER ISH; 42.5% *vs.* 6.1%; *p* < 0.001), and were frequently classified as MSI-high (MSI-H; 20.0% *vs.* 7.3%; *p* = 0.015). Cytoplasmic Akt expression was significantly higher in *PIK3CA*-mutant tumors than in *PIK3CA* wild-type tumors (immunohistochemistry [IHC] 2+ or 3+: 42.8% *vs.* 23.3%; *p* = 0.001). However, overall survival (OS) was similar between patients with *PIK3CA*-mutant tumors and those with *PIK3CA* wild-type tumors (5-year OS rate: 60.0% [mutant] *vs.* 61.3% [wild-type]; *p* = 0.944; Figure 1A).

**Table 1:** Clinicopathological characteristics of patients in cohort 1 according to their *PIK3CA* mutation status

Characteristics	*PIK3CA* wild-type	*PIK3CA* mutant	*p*-value
	(*n* = 262) (%)	(*n* = 40) (%)	
Sex			
Male	174 (66.4)	28 (70.0)	0.653
Female	88 (33.6)	12 (30.0)	
Age			
< 70 years	212 (80.9)	29 (72.5)	0.217
≥ 70 years	50 (19.1)	11 (27.5)	
Primary tumor location*			
Lower third	134 (53.8)	17 (44.7)	0.021
Middle third	71 (28.5)	7 (18.4)	
Upper third	44 (17.7)	14 (36.8)	
Pathology			
Well or moderately differentiated adenocarcinoma	98 (37.4)	8 (20.0)	0.018
Poorly differentiated adenocarcinoma	121 (46.2)	29 (72.5)	
Signet ring cell carcinoma	32 (12.2)	2 (5.0)	
Mucinous carcinoma	11 (4.2)	1 (2.5)	
Lauren classification			
Intestinal type	93 (35.5)	6 (15.0)	< 0.001
Diffuse type	143 (54.6)	23 (57.5)	
Mixed type	26 (9.9)	11 (27.5)	
T stage			
pT1/T2	91 (34.7)	6 (15.0)	0.018
pT3	85 (32.4)	13 (32.5)	
pT4	86 (32.8)	21 (52.5)	
N stage			
pN0	53 (20.2)	9 (22.5)	0.774
pN1/N2	114 (43.5)	15 (37.5)	
pN3	95 (36.3)	16 (40.0)	
Stage (by AJCC 7th edition)			
I/II	123 (46.9)	13 (32.5)	0.087
III/IV	139 (53.1)	27 (67.5)	
Lymphatic invasion			
Absent	65 (24.8)	3 (7.5)	0.015
Present	197 (75.2)	37 (92.5)	
Vascular invasion			
Absent	219 (83.6)	26 (65.0)	0.005
Present	43 (16.4)	14 (35.0)	
Perineural invasion			
Absent	121 (46.2)	11 (27.5)	0.026
Present	141 (53.8)	29 (72.5)	
Stroma reaction			
Absent	127 (48.5)	11 (27.5)	< 0.001
Desmoplasia	87 (33.2)	7 (17.5)	
Lymphoid or neutrophil	48 (18.3)	22 (55.0)	
EBER *in situ* hybridization			
Negative	246 (93.9)	23 (57.5)	< 0.001
Positive	16 (6.1)	17 (42.5)	
MSI			
MSS	243 (92.7)	32 (80.0)	0.015
MSI-H	19 (7.3)	8 (20.0)	
Gastrectomy			
Subtotal gastrectomy	194 (74.0)	21 (52.5)	0.005
Total gastrectomy	68 (26.0)	19 (47.5)	
Akt expression (cytoplasmic intensity)			
Negative	48 (20.7)	3 (8.6)	0.001
1+	130 (56.0)	17 (48.6)	
2+	49 (21.1)	9 (25.7)	
3+	5 (2.2)	6 (17.1)	

Abbreviations: AJCC = American Joint Committee on Cancer; EBER = Epstein-Barr virus-encoded small RNA.

*Patients whose disease involved the entire stomach (*n* = 15) were excluded.

The same analyses were performed for 273 patients with stage IB–III tumors who had received curative surgery. The clinical and pathological characteristics of these patients according to their *PIK3CA* mutation status are compared in Supplementary Table 2. The results obtained from these patients were almost the same as those obtained for all the patients in cohort 1. Disease-free survival (DFS) after the radical surgery was not significantly different between patients with *PIK3CA*-mutant tumors and those with *PIK3CA* wild-type tumors (5-year DFS rate: 64.8% [mutant] *vs.* 68.4% [wild-type]; *p* = 0.503; Figure 1B).

### Cohort 2

In all, 120 patients were consecutively included. The male to female ratio was 77:43, and the median age was 58 years (range, 25–88 years). All the patients in cohort 2 had stage IV disease (distant metastasis) at diagnosis and underwent palliative gastrectomy. In addition, 104 patients (86.7%) in this cohort received palliative chemotherapy after the surgery.

**Figure 2:**
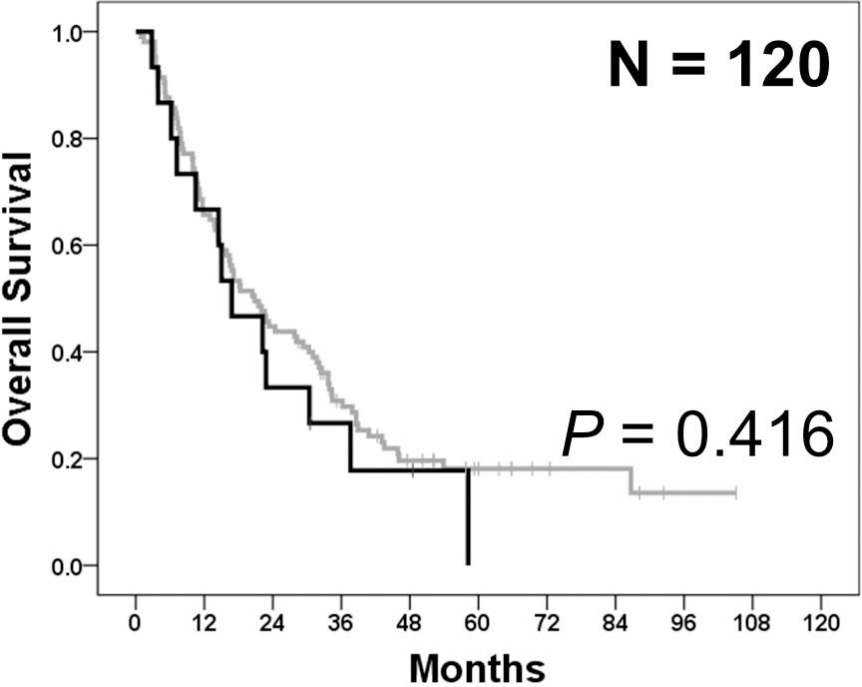
Overall survival according to *PIK3CA* mutation status in cohort 2 In cohort 2, overall survival was not significantly different between patients with *PIK3CA*-mutant tumors (black) and those with *PIK3CA* wild-type tumors (gray) (*p* = 0.416).

In all, 16 *PIK3CA* mutations were detected in 15 patients (12.5%), with E545X in 6 (5.0%) patients, H1047X in 5 (4.2%) patients, Q546X in 2 (1.7%) patients, R88Q in 2 (1.7%) patients, and E542K in 1 (0.8%) patient (Supplementary Table 1). The clinicopathological characteristics of cohort 2 according to their *PIK3CA* mutation status are compared in Table 2. *PIK3CA*-mutant tumors frequently showed vascular invasion (80.0% *vs.* 47.6%; *p* = 0.019) and MSI-H (20.0% *vs.* 3.8%; *p* = 0.041). Cytoplasmic Akt expression was significantly increased in *PIK3CA* mutant tumors (IHC 2+ or 3+: 26.7% *vs.* 11.5%; *p* = 0.018). However, primary tumor location, histological subtypes, T stage, lymphatic or perineural invasion, stromal reaction, and EBER ISH positivity were not significantly different between *PIK3CA*-mutant and *PIK3CA* wild-type tumors, which was inconsistent with the results obtained for cohort 1. OS was not significantly different between patients with *PIK3CA*-mutant tumors and those with *PIK3CA* wild-type tumors (median OS: 16.8 months [range, 7.1–26.5 months] in *PIK3CA*-mutant patients *vs.* 20.8 months [range, 14.9–26.7 months] in *PIK3CA* wild-type patients; *p* = 0.416; Figure 2).

**Table 2:** Clinicopathological characteristics of patients in cohort 2 according to their *PIK3CA* mutation status

Characteristics	*PIK3CA* wild-type	*PIK3CA* mutant	*p*-value
	(*n* = 105) (%)	(*n* = 15) (%)	
Sex			
Male	70 (66.7)	7 (46.7)	0.131
Female	35 (33.3)	8 (53.3)	
Age			
< 70 years	83 (79.0)	10 (66.7)	0.324
≥ 70 years	22 (21.0)	5 (33.3)	
Primary tumor location*			
Lower third	47 (45.2)	7 (46.7)	0.395
Middle third	28 (26.9)	6 (40.0)	
Upper third	29 (27.9)	2 (13.3)	
Pathology			
Well or moderately differentiated adenocarcinoma	28 (26.7)	4 (26.7)	0.801
Poorly differentiated adenocarcinoma	58 (55.2)	9 (60.0)	
Signet ring cell carcinoma	11 (10.5)	1 (6.7)	
Mucinous carcinoma	8 (7.6)	1 (6.7)	
Lauren classification			
Intestinal type	29 (27.6)	7 (46.7)	0.107
Diffuse type	72 (68.6)	8 (53.3)	
Mixed type	4 (3.8)	0 (0)	
T stage			
pT1/T2/T3	17 (16.2)	1 (6.7)	0.464
pT4	88 (83.8)	14 (93.3)	
N stage			
pN0/N1/N2	21 (20.0)	2 (13.3)	0.733
pN3	84 (80.0)	13 (86.7)	
Lymphatic invasion			
Absent	11 (10.5)	2 (13.3)	0.666
Present	94 (89.5)	13 (86.7)	
Vascular invasion			
Absent	55 (52.4)	3 (20.0)	0.019
Present	50 (47.6)	12 (80.0)	
Perineural invasion			
Absent	16 (15.2)	2 (13.3)	1.000
Present	89 (84.8)	13 (86.7)	
Stroma reaction			
Absent	59 (56.2)	8 (53.3)	0.868
Desmoplasia	39 (37.2)	6 (40.0)	
Lymphoid or neutrophil	7 (6.7)	1 (6.7)	
EBER *in situ* hybridization			
Negative	100 (95.2)	14 (93.3)	0.559
Positive	5 (4.8)	1 (6.7)	
MSI			
MSS	101 (96.2)	12 (80.0)	0.041
MSI-H	4 (3.8)	3 (20.0)	
Gastrectomy			
Subtotal gastrectomy	56 (53.3)	9 (60.0)	0.628
Total gastrectomy	49 (46.7)	6 (40.0)	
Akt expression (cytoplasmic intensity)			
Negative	11 (11.5)	1 (6.7)	0.018
1+	74 (77.1)	10 (66.7)	
2+	9 (9.4)	2 (13.3)	
3+	2 (2.1)	2 (13.3)	

Abbreviations: AJCC = American Joint Committee on Cancer; EBER = Epstein-Barr virus-encoded small RNA.

*Patients whose disease involved the entire stomach (*n* = 1) were excluded.

## DISCUSSION

To the best of our knowledge, this is the largest study focused on the clinical implications of *PIK3CA* mutations in GC. In addition, this is the first and largest study that shows the clinical implication of *PIK3CA* mutations in GC patients with distant metastasis. In a previous study of Fang, *et al.*, although more patients (*n* = 431) were included, they analyzed somatic mutations in the PI3K/Akt pathway genes altogether (including *AKT1, AKT2, AKT3, PTEN,* and *PIK3CA*), and did not conduct separate analyses focused on *PIK3CA* mutations [10]. Thus, their results did not provide any direct insights on the clinicopathological implications of *PIK3CA* mutations in GC patients. Moreover, they did not include GC patients with distant metastasis. In our study, the frequency of *PIK3CA* mutations was similar between 2 cohorts. In cohort 1, *PIK3CA* mutations were frequently detected in tumors located in the upper third of the stomach. In addition, these tumors significantly showed aggressive behavior such as advanced T stage; poorly differentiated histology; diffuse- or mixed-type tumors; and lymphatic, vascular, and perineural invasion. These findings were consistent when patients with stage IV GC were excluded (Supplementary Table 2). However, in cohort 2, primary tumor location and tumor behavior, except vascular invasion and MSI, were not significantly different according to *PIK3CA* mutation status.

Previous studies could not find a significant association between *PIK3CA* mutations and primary tumor location in GC [11, 12]. Recently, Fang, *et al.* analyzed mutations in the PI3K/Akt pathway by using tumor tissues from 431 patients with stage I-III GC [10]. This study showed that approximately two-third mutations in the PI3K/Akt pathway were located in *PIK3CA*. In addition, they reported that the mutations in the PI3K/Akt pathway were not significantly associated with primary tumor location. However, in intestinal-type patients, tumors with mutated PI3K/Akt pathway were less frequently located in the upper third of the stomach. In contrast, in diffuse-type patients, tumors with mutated PI3K/Akt pathway were more frequently located in the upper third of the stomach. Because mutations in the PI3K/Akt pathway located in *AKT1*, *AKT2*, *AKT3*, *PTEN*, and *PIK3CA* were analyzed altogether [10], their results did not provide any direct insights on the association between *PIK3CA* mutation status and primary tumor location. In our study, we could not find a significant association between *PIK3CA* mutations and primary tumor location according to Lauren classification (Supplementary Table 3). However, in diffuse-type patients in cohort 1, *PIK3CA*-mutant tumors tended to be more frequently located in the upper third of the stomach than *PIK3CA* wild-type tumors.

In cohort 1, *PIK3CA*-mutated tumors had significantly more aggressive features than *PIK3CA* wild-type tumors. However, in cohort 2, except vascular invasion, other aggressive characteristics in *PIK3CA*-mutated tumors in cohort 1 were not observed. Because all the patients in cohort 2 had stage IV disease, the proportion of tumors showing lymphatic, vascular, and perineural invasion was higher compared with cohort 1. Therefore, the effects of *PIK3CA* mutations on tumor aggressiveness in cohort 2 appear less distinct than those in cohort 1. The results suggest that *PIK3CA* mutations might be involved in the aggressiveness of GC in the locoregional stage, rather than in the metastatic stage. Previous studies have failed to identify the association due in part to the relatively small sample size [14–17]. Fang and colleagues showed that mutations in the PI3K/Akt pathway were more frequently present in intestinal-type tumors [10]. However, other characteristics associated with tumor aggressiveness, including lymphovascular invasion, were not significantly different according to the PI3K/Akt pathway mutation status. Barbi, *et al.* reported that although *PIK3CA*-mutant tumors were mostly in advanced T-stage, other characteristics of tumor aggressiveness were not significantly different according to *PIK3CA* mutation status [12].

Previous studies have uniformly reported that *PIK3CA* mutations are not associated with the prognosis of patients with GC [10, 12–17]. Our study demonstrated again that the *PIK3CA* mutations were not associated with poor survival outcomes in patients with GC, although *PIK3CA*-mutated tumors had more aggressive pathologic features. It is known that lymphatic, vascular, and perineural invasions, and poorly differentiated histology are related to poor prognosis [18–21], while MSI-H is a good prognostic factor in patients with GC [22–24]. Therefore, the coexistence of both good and poor prognostic factors in patients with *PIK3CA* mutations might explain the discrepancy between pathologic features and survival outcomes. In addition, it is also possible that, if *PIK3CA* mutation increases the aggressiveness of GC mainly in the locoregional stage, the mutation may not influence the prognosis of patients after appropriate radical surgery and adjuvant chemotherapy.

Mutant *PIK3CA* activates Akt pathways, which in turn promote the growth and invasion of cancer cells [7]. This mechanism has also been explored in GC [25]. In line with these results, we confirmed that *PIK3CA* mutations are associated with Akt activation in GC, thus promoting tumor aggressiveness. An aberrantly activated PI3K/Akt pathway in *PIK3CA*-mutated GC could be a potential therapeutic target in these patients. The PI3K/Akt pathway activates mammalian target of rapamycin (mTOR). An mTOR inhibitor everolimus has been investigated in patients with GC regardless of *PIK3CA* mutation status. In phase II studies, everolimus has shown promising clinical activity in patients with previously treated GC [26, 27]. However, a phase III trial GRANITE-1 failed to show the superiority of everolimus over placebo with respect to OS [28]. Nevertheless, a recent case report showed good treatment response to everolimus in a patient with GC harboring *PIK3CA* mutation and showing pS6 overexpression [29]. In addition, *in vitro* studies involving various cancer cell lines suggest that *PIK3CA* mutations are predictive markers of everolimus sensitivity [30, 31]. BKM120, a direct PIK3CA inhibitor, and BEZ234, a dual PIK3CA and mTOR inhibitor, exert pro-apoptotic effects on gastric and colorectal cancer cell lines [32]. Moreover, the anti-proliferative effects of everolimus were more potent in *PIK3CA*-mutated cell lines than in *PIK3CA* wild-type cell lines. Therefore, further clinical trials targeting PI3K/Akt pathway in GC should be conducted in selected patients such as *PIK3CA*-mutated ones, not all comers.

Our study has some limitations. First, although this is the largest study focused on the clinicopathological and prognostic implications of *PIK3CA* mutations in GC, in some analyses, the statistical power is still insufficient to draw strong conclusions due to the limited sample size. For example, a non-inferiority log rank test of OS with 262 patients with *PIK3CA* wild-type and 40 patients with *PIK3CA* mutant achieved 52.1% power at a 0.05 type I error to detect an equivalence hazard ratio of 1.50 in cohort 1, when we conducted a *post hoc* power calculation. Therefore, in further studies, a larger sample size is required to robustly validate all findings we observed. Second, since we analyzed relatively frequent and well renowned hotspot mutations in exons 1, 4, 7, 9, and 20 of the *PIK3CA* gene, the implications of relatively rare or non-hotspot *PIK3CA* mutations that were not covered in this study are still unknown. This limitation could be overcome by using the next generation sequencing methods in further studies.

In conclusion, *PIK3CA* mutations were associated with increased tumor aggressiveness, especially in locoregional disease, and Akt activation in GC. However, *PIK3CA* mutation status was not related to the prognosis of patients. Our data suggest that *PIK3CA*-mutated GC is a distinct disease entity, which might need a different therapeutic approach.

## MATERIALS AND METHODS

### Patient cohorts and tissue microarrays

In cohort 1, patients with GC who underwent curative or palliative gastrectomy at Seoul National University Bundang Hospital (SNUBH) between May 2003 and Dec. 2005 were enrolled consecutively. Patients were excluded if they had stage IA (pT1N0M0) GC according to the 7th edition of the American Joint Committee on Cancer (AJCC) staging system. In cohort 2, patients with stage IV GC (distant metastasis) who underwent palliative gastrectomy at SNUBH between Jul. 2006 and Dec. 2012 were enrolled consecutively. Tissue microarrays were constructed by isolating representative 2-mm cores from surgical specimens by an experienced gastrointestinal pathologist. Baseline demographic, pathological, and clinical data, including DFS and OS, were collected by retrospectively reviewing electronic medical records of the patients. DFS was calculated from the date of the surgery to documented disease recurrence or death from any cause. OS was calculated from the date of the surgery to death from any cause.

### PIK3CA mutation analyses by performing real-time polymerase chain reaction

All tumor samples were collected from surgical resection specimens. Hematoxylin-Eosin stained slides were reviewed by a pathologist (H.S.L.). Tumor areas were identified, and more than 1 × 1 cm area, which contained more than 60% tumor cells, was microscopically dissected. One or two 8-μm-thick formalin-fixed paraffin-embedded (FFPE) tumor tissue sections were deparaffinized. DNA was isolated using cobas^®^ DNA Sample Preparation Kit (Roche, Branchburg, NJ, USA). Concentration of the isolated DNA was measured using NanoDrop UV spectrophotometer (Thermo Fisher Scientific, Wilmington, DE, USA). The isolated DNA was diluted using DNA specimen diluent provided in cobas^®^ 4800 Mutation Test Kit (Roche) to an optimal concentration of 2 ng/μL. Amplification and detection were performed using automated cobas^®^ X480 analyzer (Roche). Real-time polymerase chain reaction (PCR) detected mutations in exons 1, 4, 7, 9, and 20 of *PIK3CA*. Results of real-time PCR are reported using the following 12 categories: (1) E542K, (2) N345K, (3) H1047X (L, R, or Y), (4) E545X (A, D, G, or K), (5) C420R, (6) G1049R, (7) Q546X (K, R, E, or L), (8) R88Q, (9) M1043I, (10) mutation not detected, (11) invalid (sample out of range/control failure), and (12) failed (hardware/software failure).

### MSI analysis

DNA was extracted from tumor tissue and corresponding non-neoplastic gastric mucosa tissue by using InstaGene Matrix (Bio-Rad Laboratories, Hercules, CA, USA). PCR was performed using fluorescent dye-labeled primers targeting five microsatellite markers, namely, BAT25, BAT26, D2S123, D5S346, and D17S250, as recommended in Bethesda guideline on MSI [33]. Fragment analysis was performed using ABI 3130 × l genetic analyzer and GeneMapper^®^ software (Applied Biosystems, Foster City, CA, USA). Tumors were classified as MSI-H when at least two of the five markers yielded novel bands, MSI-low when additional alleles were observed with one of the five markers, and microsatellite stable when all the microsatellite markers examined showed identical patterns in both tumor and normal tissues.

### EBER ISH

EBER ISH was performed using Ventana Benchmark XT (Ventana ISH iView kit, Ventana Corporation, Tucson, AZ, USA), according to manufacturer’s instructions. Briefly, 4-µm FFPE sections were deparaffinized and digested with protease II for 8 min. Next, probes were added to the samples, and the samples were denatured at 85°C for 12 min and were hybridized at 57°C for 1 h. After hybridization, the tissue samples were washed and incubated with anti-fluorescein monoclonal antibody for 20 min. Detection was performed using iView Blue Biotinylated Ig for 8 min (Ventana Corporation). Counterstaining was performed using Nuclear Fast Red II for 4 min (Ventana Corporation).

### IHC for Akt

IHC for Akt was performed using an antibody against Akt1 (dilution, 1:100; catalog no. ab32505; Abcam, Burlingame, CA, USA). Immunostaining was performed using Leica Bond-Max Automation and Leica Bond Polymer Detection Kit (Leica Biosystems, Bannockburn, IL, USA), according to the manufacturer’s recommendations. Sections were deparaffinized, and antigens were retrieved using pH 9.0 Bond Epitope Retrieval Solution 2. Slides were incubated with the primary antibody for 60 min, followed by incubation with the polymer for 8 min. After counterstaining, Akt expression in the cytoplasm of tumor cells was examined. Staining intensity was graded as follows: 0, negative; 1+, weakly positive; 2+, moderately positive; and 3+, strongly positive. In addition to staining intensity, area of positive cancer cells was scored. For statistical analysis, cytoplasmic AKT staining was regarded as positive if 5% or more cancer cells expressed it.

### Statistical analysis

Statistical analysis of categorical variables was performed using Pearson’s chi-square test, Fisher’s exact test, or linear-by-linear association, as appropriate. Median DFS and OS were calculated using Kaplan–Meier method. Survival data were compared using log rank test. All the statistical tests were two-sided, with level of significance defined at *p* < 0.05. All the analyses were performed using IBM SPSS version 22.0 (IBM, Armonk, NY, USA).

## SUPPLEMENTARY MATERIALS TABLES


