# Supplementary material for: PIK3CA mutations are associated with increased tumor aggressiveness and Akt activation in gastric cancer
Source: Oncotarget. 2017 Jun 28;8(53):90948–58. doi: 10.18632/oncotarget.18770 (PMC5710896; doi:10.18632/oncotarget.18770)
Supplement: Supplementary file 1 [file oncotarget-08-90948-s001.pdf]

PIK3CA mutations are associated with increased tumor aggressiveness and Akt activation in gastric cancer

Supplementary Materials

Supplementary Table 1: Frequency of *PIK3CA* mutations in patients in cohorts 1 and 2

*PIK3*CA mutations	Cohort 1 (%)*	Cohort 2 (%)†
E545X (A, D, G, or K)	22 (7.3)	6 (5.0)
E542K	10 (3.3)	1 (0.8)
H1047X (L, R or Y)	7 (2.3)	5 (4.2)
Q546X (K, R, E, or L)	2 (0.7)	2 (1.7)
N345K	2 (0.7)	0 (0)
R88Q	1 (0.3)	2 (1.7)
C420R	1 (0.3)	0 (0)

*Five patients had two mutations simultaneously: E542K and E545X (n = 1), E542K and H1047X (n = 1), E545X and C420R (n = 1), H1047X and R88Q (n = 1), and N345K and E545X (n = 1).

†One patient had H1047X and Q546X mutations simultaneously.

Supplementary Table 2: Clinicopathological characteristics of patients with stage IB–III gastric cancer in cohort 1 according to their *PIK3CA* mutation status

Characteristics	*PIK3CA* wild-type (n = 236) (%)	*PIK3CA* mutant (n = 37) (%)	p-value
Sex			
Male	157 (67)	27 (73)	0.437
Female	79 (33)	10 (27)	
Age			
< 70 years	191 (81)	28 (76)	0.455
≥ 70 years	45 (19)	9 (24)	
Pathology			
Well or moderately differentiated adenocarcinoma	94 (40)	8 (22)	0.029
Poorly differentiated adenocarcinoma	104 (44)	26 (70)	
Signet ring cell carcinoma	27 (11)	2 (5)	
Mucinous carcinoma	11 (5)	1 (3)	
Lauren classification			
Intestinal type	89 (38)	6 (16)	0.001
Diffuse type	124 (53)	21 (57)	
Mixed type	23 (10)	10 (27)	
Primary tumor location*			
Lower third	126 (55)	16 (44)	0.033
Middle third	63 (28)	7 (19)	
Upper third	40 (17)	13 (36)	
T stage			
pT1/T2	91 (39)	6 (16)	0.009
pT3	81 (34)	13 (35)	
pT4	64 (27)	18 (49)	
N stage			
pN0	51 (22)	9 (24)	0.788
pN1/N2	110 (47)	15 (41)	
pN3	75 (32)	13 (35)	
Stage (by AJCC 7th edition)			
I	51 (22)	5 (14)	0.072
II	72 (31)	8 (22)	
III	113 (48)	24 (65)	
Lymphatic invasion			
Absent	62 (26)	3 (8)	0.016
Present	174 (74)	34 (92)	
Vascular invasion			
Absent	207 (88)	25 (68)	0.001
Present	29 (12)	12 (32)	
Neural invasion			
Absent	117 (50)	11 (30)	0.024
Present	119 (50)	26 (70)	
Stroma reaction			
Absent	113 (48)	10 (27)	< 0.001
Desmoplasia	77 (33)	7 (19)	
Lymphoid or neutrophil	46 (19)	20 (54)	
EBER *in situ* hybridization			
Negative	220 (93)	21 (57)	< 0.001
Positive	16 (7)	16 (43)	
MSI			
MSS	218 (92)	30 (81)	0.058
MSI-H	18 (8)	7 (19)	
Gastrectomy			
Subtotal gastrectomy	179 (76)	20 (54)	0.006
Total gastrectomy	57 (24)	17 (46)	
Akt expression (cytoplasmic intensity)			
Negative	43 (20)	3 (9)	0.002
1+	115 (55)	16 (48)	
2+	47 (22)	8 (24)	
3+	5 (2)	6 (18)	

Abbreviations: AJCC = American Joint Committee on Cancer; EBER = Epstein-Barr virus-encoded small RNA.
*Patients whose disease involved the entire stomach (n = 8) were excluded.

Supplementary Table 3: Association between *PIK3CA* mutations and primary tumor location according to Lauren classification in patients in cohort 1

(A) Intestinal type (n = 99)

Primary tumor location*	*PIK3CA* wild-type (n = 93) (%)	*PIK3CA* mutant (n = 6) (%)	p-value
Lower third	55 (59.1)	4 (66.7)	
Middle third	23 (24.7)	1 (16.7)	1.000
Upper third	15 (16.1)	1 (16.7)	

(B) Diffuse type (n = 153)

Primary tumor location*	*PIK3CA* wild-type (n = 132) (%)	*PIK3CA* mutant (n = 21) (%)	p-value
Lower third	62 (47.0)	9 (42.9)	
Middle third	42 (31.8)	4 (19.0)	0.227[†]
Upper third	28 (21.2)	8 (38.1)	

[*]Patients whose primary tumor involved the entire stomach were excluded.

[†]When primary tumor location was categorized into two groups (upper vs. middle/lower), the p-value was 0.090 by chi-square test.
